# Development and usability evaluation of a mHealth application for albinism self-management

**DOI:** 10.1186/s12911-023-02202-7

**Published:** 2023-06-13

**Authors:** Saman Mortezaei, Reza Rabiei, Farkhondeh Asadi, Hassan Emami

**Affiliations:** Department of Health Information Technology and Management, School of Allied Medical Sciences, Shahid Beheshti University of Medical Sciences, Tehran, Iran

**Keywords:** Albinism, mHealth, Self-management, Usability testing

## Abstract

**Background:**

Reduced or absence of melanin poses physical, social, and psychological challenges to individuals with albinism. Mobile health (mHealth) applications have the potential to improve the accessibility of information and services while reducing time and costs. This study aimed to develop and evaluate a mHealth application for self-management of albinism.

**Methods:**

This applied study was conducted in two stages (development and evaluation) in 2022. Initially, the functional requirements were determined, and the conceptual model of the application was then developed using Microsoft Visio 2021. In the second phase, the application was evaluated using the Mobile Application Usability Questionnaire (MAUQ) involving patients with albinism to reflect their views on the usability of the application.

**Results:**

The key capabilities of the application included: reminders, alerts, educational content, useful links, storage and exchange of images of skin lesions, specialist finder, and notifications for albinism-relevant events. Twenty-one users with albinism participated in the usability testing of the application. The users were predominantly satisfied with the application (5.53 ± 1.10; Max: 7.00).

**Conclusions:**

The findings of this study suggest that the developed mobile application could assist individuals with albinism to effectively manage their condition by considering the users’ requirements and services that the application should deliver.

**Supplementary Information:**

The online version contains supplementary material available at 10.1186/s12911-023-02202-7.

## Background

Albinism refers to a group of hereditary disorders that cause reduced or lack of melanin pigment synthesis. Melanin is naturally produced by melanocytes in skin, hair, and eyes. Depending on the involvement of the eyes, albinism is classified into two main types: ocular albinism and oculocutaneous albinism [1–4]. In addition, high phenotypic variation in albinism causes patients to fall into a wide range of conditions, from complete to partial albinism [5].

Different rates of albinism prevalence have been reported across the world. It is estimated that albinism has a global incidence of approximately one case per 17,000 people. However, higher rates of one case per 5,000 population and even one case per 1,000 population in some particular groups have been reported [2, 6].

Skin conditions, such as frequent sunburns and actinic keratosis caused by exposure to solar UV radiation can potentially lead to malignancies if left untreated. In addition, light skin is considered one of the most important risk factors for developing life-threatening malignancies including melanoma and squamous cell carcinoma (SCC). In addition to skin complications, almost all types of albinism are associated with a degree of visual impairment. The majority of people with albinism have a low visual acuity and fall into the visually impaired group [7, 8]. Iris translucency, retinal hypopigmentation, foveal hypoplasia, refractive errors, reduced visual acuity, and in some cases, color blindness are among the visual disorders in people with albinism [2, 9–11]. Moreover, people with albinism face social and psychological challenges. Depression and generalized anxiety disorder are two cases of psychological problems observed in people with albinism [12]. Limited awareness has caused albinism to be regarded as a negative trait, and society’s view of people with albinism might be inappropriate, or in some cases, it is accompanied by pity. These factors have caused people with albinism to withdraw from social situations and experience difficulties in education, employment and marriage [13]. Hence, paying attention to the social and psychological aspects of albinism is also of great importance in the management of this disorder.

Like many other genetic disorders, albinism is incurable and its management is mainly based on performing daily activities that maintain and improve the well-being of individuals with albinism [5, 14]. Gilchrest suggests that the use of clothes with sufficient coverage and sunscreen with a high protection factor could be effective measures for maintaining skin health [15]. Unlike skin conditions, control, or prevention of visual problems in albinism can be easily achieved. Therefore, patients may require special equipment to perform their daily activities. The management of depression and anxiety, on the other hand, may be performed differently. Pharmacotherapy and psychotherapy sessions alone, or in some cases, combined with other approaches are recommended in this respect [16, 17].

In recent years, mobile health (mHealth), has been used in various fields of medicine, including education, care, prevention, and treatment [18, 19]. With respect to skin care, mHealth applications could provide services including self-assessment, treatment adherence, reminders, nutritional and health recommendations, as well as alarms for sun protection [20]. There are also store-forward imaging applications for skin care that provide specialists with information about skin lesions along with patient information. To manage and improve the daily activities of people with visual impairments, various applications are supported with tools for color recognition, object recognition, navigation, reading, and text writing [21]. Applications developed for the management of psychological problems provide services such as psychoeducation, social support, follow-up treatment and healthcare advice [22].

Considering the importance of self-management in individuals with albinism as well as the wide variety of services that could be offered by mobile applications [20], developing applications for self-management of this condition is important. It is also necessary for developers to always take the needs of the given target group into consideration and involve different stakeholders in the development of applications. Unfortunately, despite the numerous problems that individuals with albinism experience on a daily basis, studies addressing the use of mobile applications in the self-management of albinism are limited. In addition, existing mobile applications have either poor performance or poor usability, which can be attributed to the lack or little participation of experts and users in the development of these applications [23]. To our knowledge, almost all mobile applications developed for albinism at the time of conducting this research aimed mainly to raise individuals’ awareness of this disorder or to share information with specialists. Furthermore, other facets of self-management, including the social and psychological aspects, have been overlooked. On that account, the present study aimed to develop and evaluate an mHealth application for albinism self-management.

## Materials and methods

The current applied-developmental study was carried out in two stages in 2022.

### Application development

Initially, a literature search was conducted in May 2022 on PubMed, Web of Science, Scopus, Cochrane, and IEEE Xplore using the following MeSH terms: albinism, self-care, self-management, and mobile health. The search was limited to studies published between January 1, 2017 and April 31, 2022. The literature review aimed to identify key requirements of the application that should be addressed in the development phase to obtain a better understanding of the albinism and challenges faced by people with this condition, and to gain insight into the information content of the system. Subsequently, an expert panel was conducted with the participation of the director of the Iranian Association of Albinism, two dermatologists, two psychologists, and two ophthalmologists, to discuss the content of the application and its required services. The inclusion criteria for the six specialists were as follows: having at least five years of experience in their field and being a faculty member at one of the medical universities in Tehran. The director of the Iranian Association of Albinism had more than five years of experience in this field. At this stage, participants were introduced to the research team by the Albinism Association. Due to time limitations of the panelists and challenges of setting a mutually convenient time, we held only one round of focus group, in which one researcher (RR) moderated the session and led the discussions, and the other (SM) acted as a co-moderator, overseeing note taking and facilitating the presentation. The functional requirements extracted from the literature were presented to the panel. Each requirement was presented and discussed separately in the panel. The information content of the application was also a matter of discussion in the panel.

Based on the findings from the panel of experts, the conceptual model of the application, which consisted of the functional, structural, and behavioral models, was designed using Microsoft Visio 2021. The model was then verified by two experts in the field of medical informatics to ensure that the requirements of the application were appropriately addressed. The application was then programmed in Java and Kotlin languages using Android Studio 2021 and subsequently tested and debugged.

### Application evaluation

At this stage, the usability of the application was evaluated based on the participation of individuals with albinism. The tool used in the evaluation of the application was the mHealth App Usability Questionnaire developed by Zhou et al. [24], set into three sections: ease of use (5 criteria), user interface and satisfaction (7 criteria) and usefulness (6 criteria) on a 7-point Likert scale ranging from completely disagree (1 point) to completely agree (7 points). The questionnaire was translated into Persian as there was no Persian version of this tool available. The translated questionnaire was then translated back into English by two experienced English translators to ensure the cross-cultural adaptation. The face validity of the questionnaire was evaluated by two medical informatics experts, and its reliability was confirmed in previous studies [24–26]. The reliability of the questionnaire was re-examined and the internal correlation coefficient of the questions was calculated (Cronbach’s alpha = 0.94). Participants were involved in the study on a voluntary basis. The sample size was based on the number of available participants and their willingness to take part in the study. The inclusion criteria were as follows: willingness to take part in the study, age between 13 and 60 years, being a member of the Iranian Association of Albinism, and the use of smartphone. Participants were provided with necessary explanations about the aim of the study and instructions about the application, and they were assured of the confidentiality of the collected data. The collected data were analyzed using SPSS 26, and by calculating Mean and Standard Deviation.

To facilitate the reporting of the results, we calculated the quarters for the total mean value (7.00). The range of mean values for each quarter was as follows: 0-1.75 (poor), 1.76–3.50 (moderate), 3.51–5.25 (good), and 5.26-7 (very good).

## Findings

### Findings related to application development

**Fig. 1:**
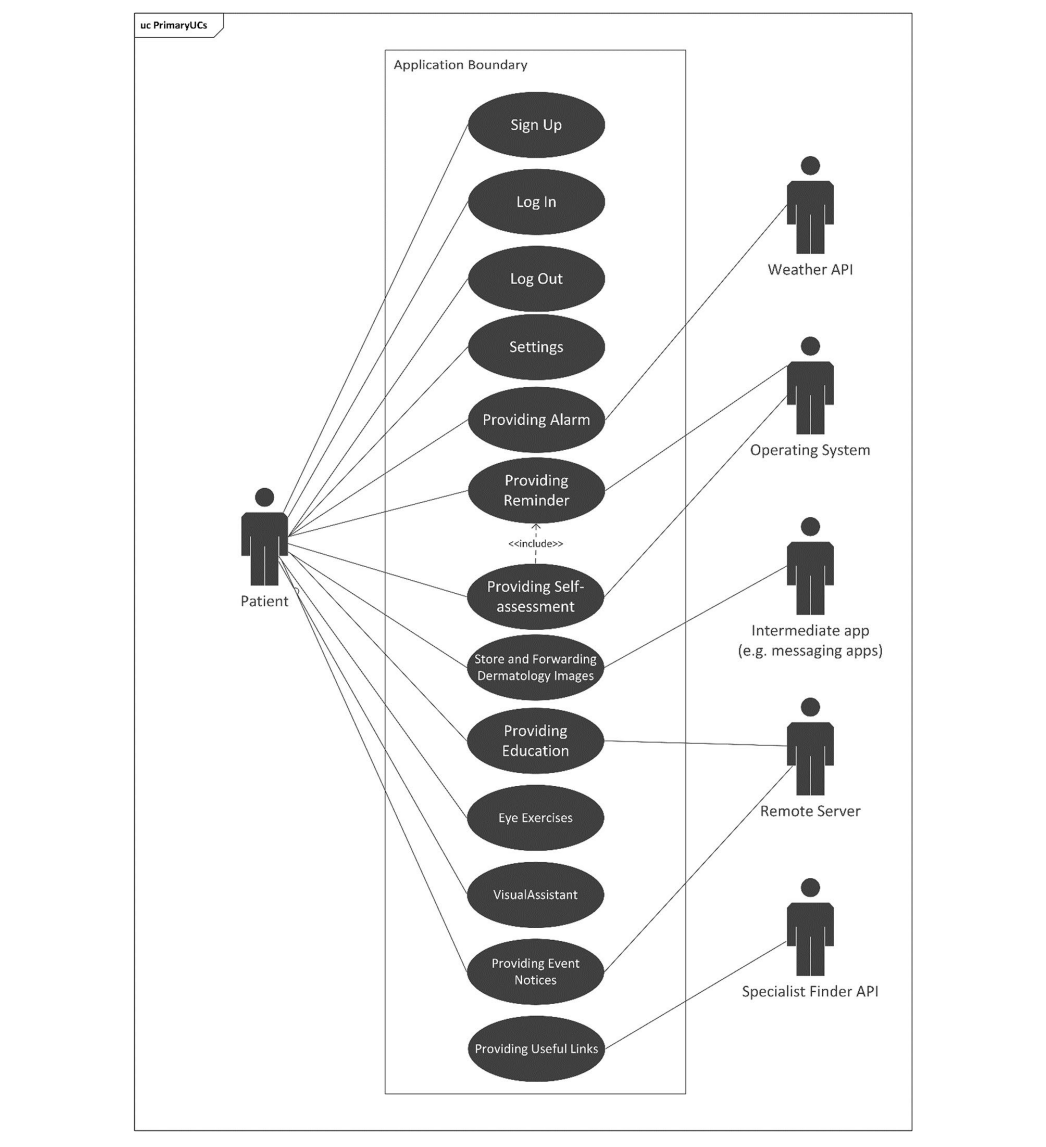
Use-case diagrams of the application. Application Programming Interface (API)

The functional requirements initially extracted from the literature review were presented in the expert panels to ensure that the required functions were applied in the design of the application (Table 1). The overall use-case diagram of the application is presented in Fig. 1. The main services of the application included: alarms, reminders, storage and exchange of skin lesion images, education, eye exercises, notification of related events, specialist finder, and useful links. The Class Diagram of the application was provided in Appendix A.

The expert panel participants included three females (42.9%) and four males (57.1%). Their age range was categorized into two groups of 40–50 (n = 4, 57.1%) and 51–60 (n = 3, 42.9%). In addition, two participants (28.6%) had less than 11 years of work experience, and the rest (n = 5, 71.4%) had work experience of more than 11 years. All requirements were approved by the panel and one requirement (Events Notification) was suggested to be added to the list after discussion and approval by the panel members.

**Table 1:** Functional requirements of the application

	Functional Requirement	Description
1	Signing up	The application should enable users to create an account in the application by setting a username and password
2	Log-in	The application should enable users to enter the application using his/her username and password
3	Log-out	The application should enable users to log out of the application
4	Settings	The application should enable users to adjust the settings of the application, including the notification sound, application appearance and his/her current location (to receive weather information)
5	Providing alerts	The application should inform the user of harmful conditions by creating alerts.
6	Providing reminders	The application should remind the user to take medications and see his/her health professional
7	Store and forwarding skin lesion images	The application should enable users to save and classify images of his/her skin lesions and send them to his/her health professional
8	Providing education related to skin, vision and mental health	The application should improve the user’s awareness of their health conditions by providing relevant educational materials
9	Self-assessment of mental health	The application should enable the user to assess his/her psychological status and create assessment reports
10	Eye exercises	The application should provide the users with eye exercises
11	Events Notification	The application should provide the user with events related to albinism
12	Specialist Finder	The application should help the user find relevant specialists
13	Useful links	The application should provide the user with useful links

**Fig. 2:**
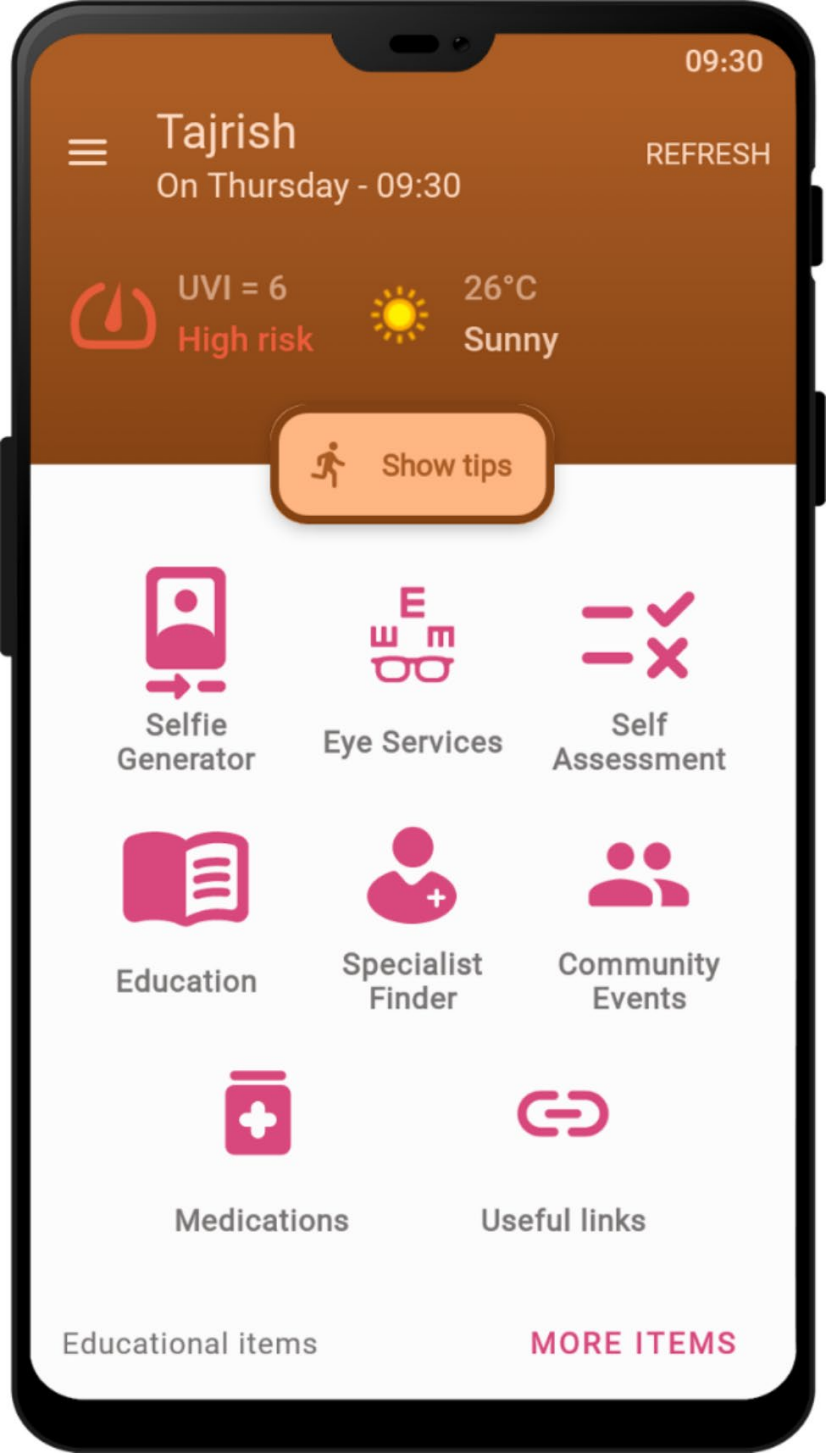
Main menu of the application

**Fig. 3:**
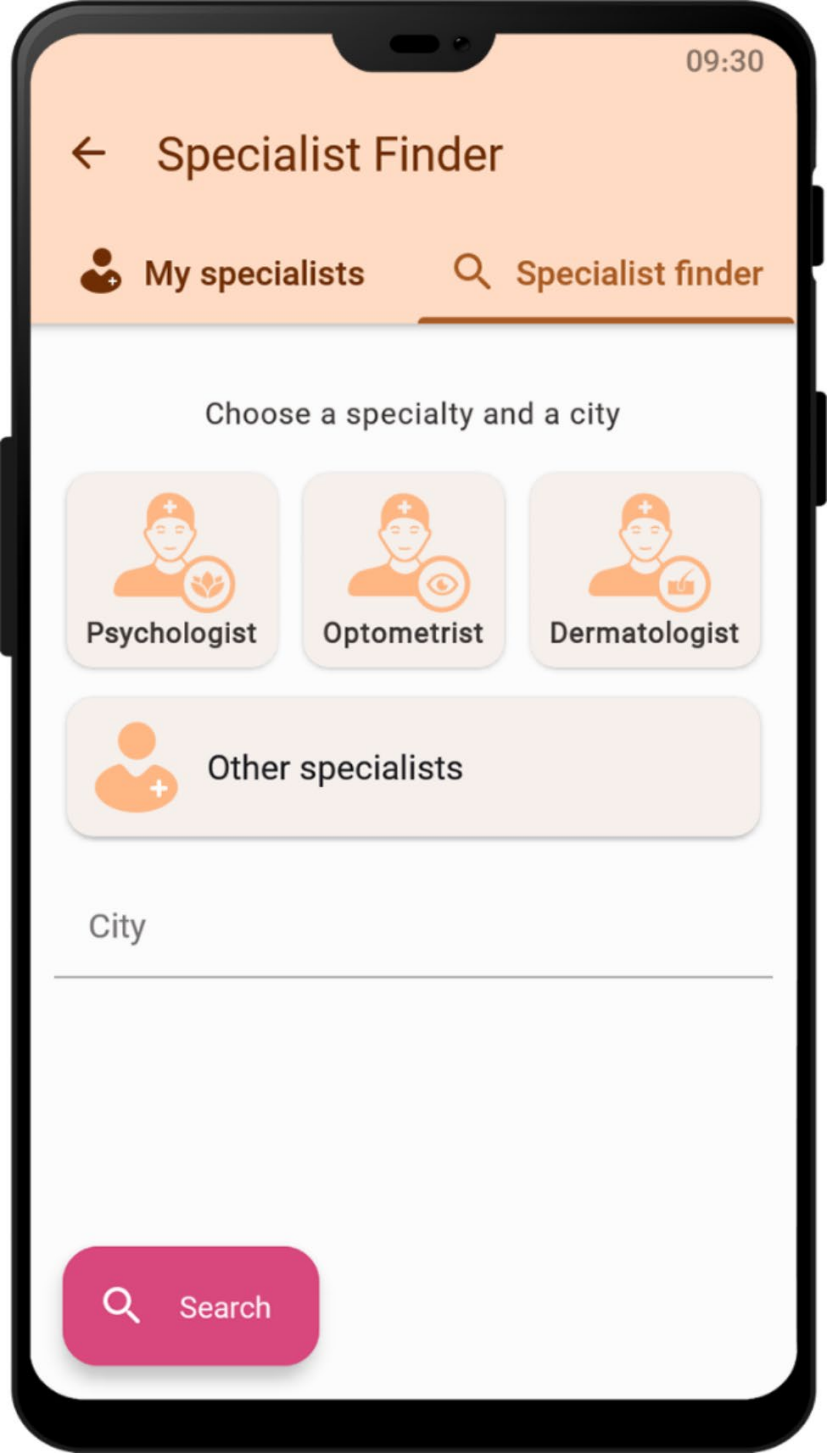
Specialist finder screen

**Fig. 4:**
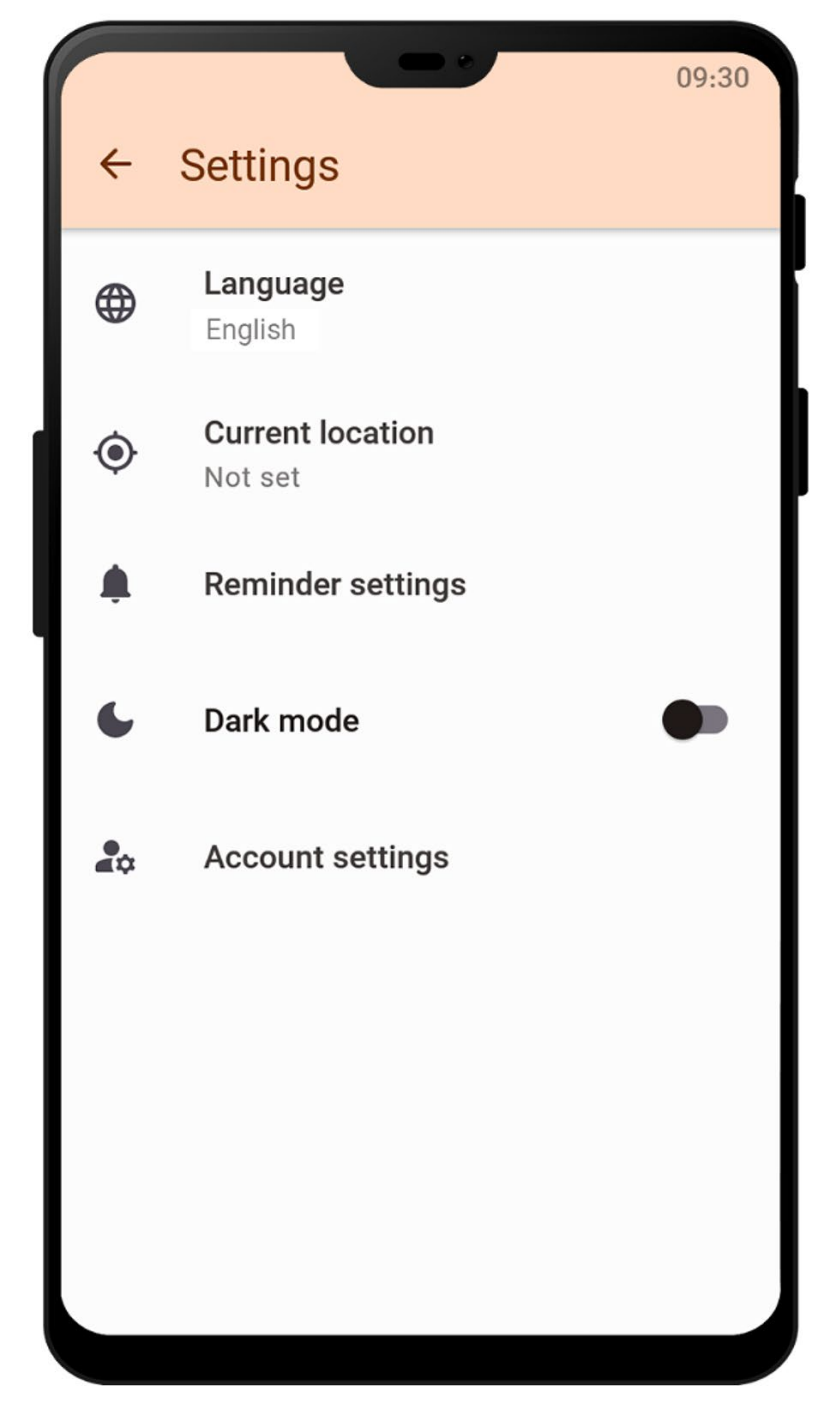
Settings screen

On the main menu of the application, weather information and UV index are displayed based on the user’s current location. In the event of the UV index being in the harmful range, the user is warned using an automated alert notification, and related recommendations are provided accordingly (Fig. 2). Figure 3 shows the screen related to the specialist finder service. This service enables users to search for specialists based on their specialty and location (Fig. 3). In the settings section (Fig. 4), users are provided with the options to change settings related to the location, reminders, account, and the appearance of the application based on their preferences. Educational service of the application included educational materials related to mental health, skin care, and eye care in the format of text and image. It is also possible to add audio and video clips to the educational content of the application.

### Findings related to application evaluation

Twenty-one participants were involved in the evaluation of the application. The average age of the participants was 29.52 ± 9.95 years, with a minimum of 13 years and a maximum of 51 years. The education level of the participants ranged from primary education to doctorate, with bachelor’s degree having the highest frequency (Table 2).

**Table 2:** Demographic details of participants invloved in the evaluation of the application (N = 21)

Variable		N (%)
Gender	Male	8 (38.10)
	Female	13 (61.90)
Age	< 18	3 (14.30)
	18–28	7 (33.30)
	29–50	10 (47.60)
	50 +	1 (4.80)
Education	High school	5 (23.80)
	Diploma	2 (9.50)
	Associate Degree	0 (0.00)
	Bachelor’s Degree	9 (42.90)
	Master’s Degree	4 (19.00)
	Doctorate	1 (4.80)

The total mean score for the usability of the application was 5.53 ± 1.10. Among the usability criteria, the highest mean score was assigned to the ease of use, with a mean score of 6.01 ± 0.89. The mean score of 5.51 ± 1.45 was obtained for the user interface and satisfaction, followed by a mean score of 5.16 ± 1.23 for the usefulness of the application (Table 3).

**Table 3:** Descriptive statistics of the mHealth application usability questionnaire main criteria (N = 21)

Dimensions of Usability	Mean ± SD (Max = 7.00)	Level
Ease of Use	6.01 ± 0.89	Very Good
Interface and Satisfaction	5.51 ± 1.45	Very Good
Usefulness	5.16 ± 1.23	Good
Total	5.53 ± 1.10	Very Good

Standard Deviation (SD)

## Discussion

Given the vulnerability of individuals with albinism both physically and psychologically, and the crucial role of self-management of their condition, an application was developed and evaluated to facilitate self-management of skin, vision, and psychological complications in individuals with albinism. According to the findings, the overall level of usability of the application was assigned as very good, and among the three dimensions used to measure the usability of the application, the “usefulness” obtained the lowest mean score. A possible reason for this finding could be the short interval between the use of the application and its evaluation which was insufficient to measure the impact of the application on health, well-being and effective management of health conditions. In their study, Coups and Ritterband indicated that services such as treatment adherence, sun protection, and self-assessment could help improve, control and monitor disease behaviors and symptoms for skin care [20]. In the current study, the application has the capability of supporting users in monitoring their skin lesions over time and sharing images of their lesions with their specialist. In addition, providing alarms to protect users against solar radiation based on their current location was one of the services provided in the application. These services were similarly provided in a study by Buller et al., who developed a mobile application to protect the skin from harmful solar radiation. In their application, recommendations for skin care were provided based on the time of the day, UV index, location, and phenotypic characteristics of the user [27]. The findings of Buller et al.‘s study showed that the use of mobile applications of this type could effectively reduce the risks of sun exposure [28]. The findings of the current study and those reported in the aforementioned studies suggest the role of mHealth applications in skin protection that could help to minimize the consequences of skin problems.

In a study by Einollahi et al., in which the possibilities and content of dermatology applications were examined, educational content was introduced as an effective service in self-management of skin problems [29]. In another study by Karthikeyan et al., the provision of educational content was considered an important feature in eye-related health applications that aimed at helping patients better understand their condition [30]. In this regard, providing educational contents related to albinism self-management is a main service in the present study, which was implemented based on relevant literature. However, exploring users’ requirements, including educational needs, is key to developing educational content [31, 32], which was not addressed in the current study due to limited access to individuals with albinism during requirement analysis and design of the application.

In a study by Hogarty et al., in which different smartphone applications in optometry were examined, the reminder function of the applications was introduced as a key service for treatment adherence [33]. In the current study, there was a reminder function for taking medications as well as setting up appointments with health professionals, which could put patients in better control of their condition.

The use of mobile applications for the treatment of visual problems is yet another important aspect of mHealth applications. If developed based on scientific evidence, users’ requirements, and specialists’ involvement, these applications could be effective in treating some visual problems, including lazy eye in adults [34]. In the current study, after reviewing the literature, several visual exercises were set in the application to help people with albinism better manage conditions such as lazy eye.

In addition, there was a service for self-assessment of mental health in the current study based upon which users are periodically reminded to perform depression and generalized anxiety self-assessments. Users can also observe their assessment history over time and share it with their specialist. In a study by Areàn et al., which aimed to study the mobile technologies in mental health assessment, patient-generated data through self-assessments was considered an important service that helped healthcare professionals in more efficient service delivery [35].

In the current study, the results of the evaluation showed that the users were, to a great extent, satisfied with the application (5.53 ± 1.10). Among the three main usability criteria, users were most satisfied with the ease of use of the application (6.01 ± 0.89), which could be related to features such as the learnability of the application and moving between different screens. Ease of use refers to aspects including the consistency of navigation and ease of learning that enable users to have more efficient use of the application [24]. Similarly, the results of a study by Meryk et al., who used MAUQ to evaluate the usability of an mHealth application showed that ease of use obtained the highest mean score along with satisfaction [36]. These findings are also in line with the study by Bin-Azhar and Dhillon, who suggested that ease of use is an important factor in the effective use of mHealth applications in the self-management of health problems [37].

A positive point of the current study could be its specific focus on developing a mobile application for individuals with albinism, taking eye care, skin care, and mental health into consideration. In addition, the functional requirements of the application that initially extracted from the literature were then discussed in the focus group session, and these two steps led to consideration of the key services provided in the application. However, there were some limitations to the current study. First, the application was developed for devices running the Android operating system, which affected the use of the developed application for devices with other operating systems such as iOS. In addition, the duration of the study, especially in the evaluation phase, did not permit the evaluation of the application in terms of impacts, such as meaningful behavior or behavior change and visual improvements. Due to the specific nature of this disorder and its complexities, our two rounds of recruitment attempt to involve individuals with albinism to explore their needs failed before designing the system. The Iranian Association of Albinism played an active role in the usability evaluation of the application, involving 21 volunteers. Nevertheless, the sample size was limited; therefore, further evaluation of the application in terms of usability and impacts over a longer time span and broader population is required.

## Conclusion

The mobile application for albinism self-management, developed and tested in the current study, has the potential to be used as a complement to other medical interventions, such as pharmacotherapy and psychological treatments while saving time and money. Ease of use and user satisfaction obtained the highest mean score and were considered as very good, whereas the usefulness of the application was regarded as good. These findings suggest the pivotal role of end-user involvement in the design and development of mHealth applications and in improving usability aspects, including usefulness. In addition, over time and with the utilization of the application, further evaluation is suggested to improve the application, particularly in relation to its clinical implications and usefulness.

## Electronic supplementary material

Below is the link to the electronic supplementary material.

Supplementary Material 1

## Data Availability

The data generated, analysed and used during the current study are not publicly available due to Shahid Beheshti University of Medical Sciences policy, but are available from the corresponding author upon the reasonable request.
