# Supplementary figures and images for: Development and usability evaluation of a mHealth application for albinism self-management
Source: BMC Med Inform Decis Mak. 2023 Jun 13;23:106. doi: 10.1186/s12911-023-02202-7 (PMC10262590; doi:10.1186/s12911-023-02202-7)

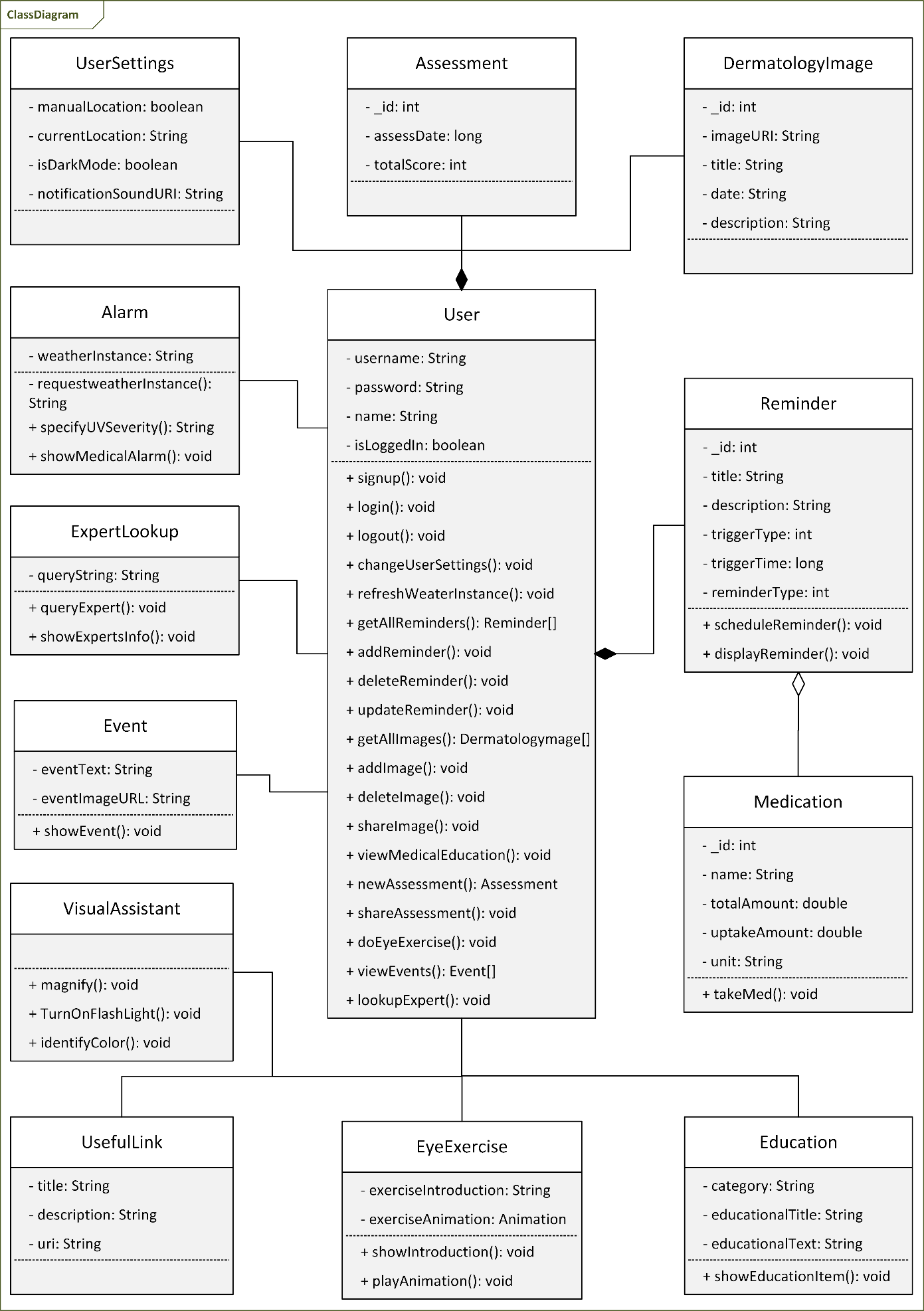
Appendix 1. Class diagram of the application.

Supplement: Supplementary file 1 — Supplementary Material 1 [file 12911_2023_2202_MOESM1_ESM.docx]
